# Global Technical Variations in Roux-en-Y Gastric Bypass: A Worldwide Survey Among IFSO Members

**DOI:** 10.1007/s11695-026-08724-z

**Published:** 2026-05-23

**Authors:** Mohamed Hany, Mohamed H. Zidan, Hashem Altabbaa, Ahmed Amgad, Hazem Al-Momani, Ahmed Mosaad, Aya Bessa, Fouad Hanna, Nour Zayed, Ahmed Youssef Hassan, Omar Badawy, Omar Heih, Hassan El-Masry, Shorouk Mourad, Ahmed El-Shamarka, Marwan Emad Abdou, Anwar Ashraf Abouelnasr, Mostafa Refaie Elkeleny, Bart Torensma, Mohamed Alhashash, Hebaallah Mohamed Zaki Jaheen, Ahmed Abokhozima, TRPL-IFSO Task Force, Abdelhdi Alhawarat, Abdelrahman M. Tawfik, Ashkan Akbarpour, Fatmaelzahraa Abdelfattah, Hesham Mohamed, Hisham Sharaf, Islam Hussien, Mahmoud Eissa, Mohamed Al Sayed, Menahtallah Mohsen, Merna Mourad, Almoatazbellah Anwar Attalla, Nour Eldein Saad, Nour Lebda, Nouran Keshk, Yousef Romia, RYGB GLOBAL consortium, Abd-Elfattah Kalmoush, Abdullah Almunifi, Abdelrahman Nimeri, Adel Abou-mrad, Admar concon-Filho, Adrianus Luijten, Ahmed Guirat, Ala Wafa, Aleksandr Neimark, Aleksei A. Botov, Alex Craven, Alexandra Regina Szewczyk, Alexandre Haumann, Alexandre Henrard, Alvaro Gonzalez, Amanda Belluzzi, Ana Carolina da Costa Mello Moreira, Ana Paulina Pimienta Sosa, André Lázaro, Andres Alban Rivas, Andrés Hanssen, Andrés Muñoz Mora, Andres Ospina, Andrew G. Robertson, Angelo Bustani Loss, Angelo Iossa, Anil Ergin, Anna Casajoana, Antonio Alvigi, Arshad Ali, Arut Mezhunts, Aykhan Abbasov, Benjamin Clapp, Benjamin Robert Logan Wheeler, Benoit Seydel, Bernard Majerus, Bruno Ziade Gil, Caio Cesar Lopes Borga, Camilo Ortiz, Carlo Nagliati, Carlos A. Casalnuovo, Carlos Enrique, Carlos Madalosso, Carlos Guzmán, Carmen Balagué, Carlos J. Pérez-Padrón, Carolina Isabella Alexandra Koehler, Cássio Padilha Rubert, Celso Afonso Nabais, Cem Emir Guldogan, César Ernesto Perez, Chakib Ahmed Abbadi, Christian Reetz, Constanza Ballesta Ferrer, Cosimo Saviello, Cristian Eugeniu Boru, Daniel Gero, Daniel M. Felsenreich, Dario Raglione, David I. Robertson, David Moszkowicz, Eduardo Domínguez-Adame, Elias Chousleb, Elias Sdralis, Ellen Deleus, Emanuele Soricelli, Enric Fernández Alsina, Ernesto Jesús Barzola Navarro, Everton Cazzo, Evgeny Zorin, Farah A. Husain, Fernando de Barros, Fernando Pilla, Fernando Zacarias De Souza, Francisco Lennyn Alvarenga, Francisco Tustumi, Frank Benedix, Frédéric Borel, Frederik Lecot, Frits Berends, Georgios Peros, Geraud Tuyeras, Gianluca De Santo, Gil Faria, Gilberto Marcano, Giovanna Pavone, Giovanni Lezoche, Giuliano Sarro, Goran Marjanovic, Guillaume Pourcher, Gustavo Adolfo De Paz, Hamilton Barbosa, Hannah Pflieger, Harald Tigges, Hermann Nehoda, Hernando Javier Forero, Hosam Mohamed Elghadban, Humberto Jimenez, Ida Francesca Gallo, Ismael Diez del Val, Ivaylo Tzvetkov, Jaime Cepeda, Jan Apers, Javier Eduardo Acuña, Javier Manrique, Javier Pardo, Jean Pierre Vergnaud, Jean-Philippe Goreux, Jélis Arenas Pimentel, Jenny Baca, Jesús Alberto De la Rosa, Jesús Vakl, João Caetano Dallegrave Marchesini, Johanan David Davila Castro, Johannes Zacherl, Jorge Esmeral, Jose Enrique Gutierrez, Jose Luis Cruz Vigo, José Pedro de Almeida Pinto, Juan Castilla, Juan Daniel Rodriguez mutis, Julio Cezar Cechinel Filho, Julio Alberto Garcia Barco, Jumaev Nozim Adxamovich, Kenneth Copperwheat, Klaus Erich Gerauer, Koba Sakhechidze, Kurt Devroe, Laurent Genser, Leonardo Bortolotti, Leopa Nicoleta, Levon N. Grigoryan, Lourenilson Jose de Souza, Lucas Bojanini Acevedo, Luis Rafael Level Córdova, Luiz Alfredo D’ Almeida, Mahdi Alemrajabi, Maksims Mukāns, Manish Khaitan, Manuel Garcia, Marc J. Legrand, Marc J. Van Det, Maria Antonieta Barrera, María Asunción Acosta Mérida, Mariano Palermo, Mario Alfonso-Blaschke, Marjori Román, Marta Cuadrado-Ayuso, Martino Guenzi, Masoud Rezvani, Massimiliano Di Paola, Matthieu Poussier, Mehmet Celal Kızılkaya, Michael Talbot, Michel Suter, Michel Vix, Michele Podetta, Miguel Onofre Maia Domingues, Mirko Otto, Mirto Foletto, Misha Luyer, Mohamed Mosaad Kandel, Mohamed Omar Fathi Ammar, Mohammad Kermansaravi, Mohanarajah Silvarajah, Mohey Elbanna, Montse Adell Trapé, Mostafa Mohamed Gamal, Mostafa Nagy, Muhammed Taha Demirpolat, Muwaffaq Telfah, Nicholas Cocco, Nicola Tartaglia, Yanikck Nijs, Nikolaos Pararas, Nuru Bayramov, Oktyabr Teshaev, Omar M. Ghanem, Oscar Mauricio Gomez, Otto Jaime Montoya Tobar, Pablo Arago-Chofre, Panagiotis Lainas, Paolo Gentileschi, Patrick Moore, Patrick Téoule, Patwinder Gill, Paula Franczak, Pedro Monsalve Trejo, Philipp Beckerhinn, Piotr Major, Professor Chetan Parmar, Raffaele Galleano, Ramen Goel, Raquel Alfonso, Rob Snoekx, Roberto Coelho Netto Da Cunha Costa, Roberto Esquivel David, Rodrigo Strobel, Romano Schneider, Ronald S.L. Liem, Ronnal Vargas Cordova, Rossana Daniela Berta, Rossella D′ Alessio, Rui José Silva Ribeiro, Ruth Blackham, Saeed Ali Alsareii, Safwan Abdulrahman Taha Almaatoq, Santiago Gomez, Samer Mattar, Sergio Carandina, Sergio Sitta, Sergio O. Aparicio, Sergio Rodrigo del Valle Ruiz, Shahab Shahabi Shahmiri, Silvana Leanza, Sjaak Pouwels, Sofie Viskens, Sonja Chiappetta, Stefano Olmi, Suhaib Ahmad, Suleiman Naji, Javier T. Birriel, Talal Kamel Khewater, Therese Reinstaller, Thomas W Frick, Tom Wiggins, Tristan Greilsamer, Umut Riza Gunduz, Valdemir José Alegre Salles, Valerio Girardi, Vasileios Charalampakis, Victor Lunau Barcellos, Vincenzo Borrelli, Wah Yang, Yi Chen, Yves Borbély, Zdenko Boras, Zilvinas Dambrauskas, Zsolt Bodnar, Rodolfo J. Oviedo

**Affiliations:** 1 Department of Surgery, Alexandria Research Institute, Alexandria University, Alexandria, Egypt; 2 Madina Women’s Hospital, Alexandria, Egypt; 3 Alexandria University, Alexandria, Egypt; 4 The Research Papyrus Lab, Alexandria, Egypt; 5 Al-Basheer-Hospital, Amman, Jordan; 6 Helwan University, Cairo, Egypt; 7 NMC Royal Khalifa Hospital, Abu Dhabi, United Arab Emirates; 8 Northumbria Healthcare NHS Foundation Trust, North Shields, UK; 9 Cairo University, Giza, Egypt; 10 Tanta University, Tanta, Egypt; 11 Faculty of Medicine, Palestine Polytechnic University, Hebron, Palestinian Territory; 12 El-Ekbal Hospital, Alexandria, Egypt; 13 Clinical Epidemiology, Erasmus MC, Rotterdam, Netherlands; 14 Blackpool Victoria Hospital, Blackpool, UK; 15 Department of Surgery, Nacogdoches Medical Center, Texas, USA; 16 University of Houston, Houston, USA

**Keywords:** Roux-en-Y gastric bypass, Metabolic and bariatric surgery, Technical variation, Gastric pouch configuration, Limb length variability, Ring augmentation

## Abstract

**Background:**

Roux-en-Y gastric bypass (RYGB) is a widely performed metabolic and bariatric surgery (MBS) procedure with proven efficacy. However, significant variability exists in its technical execution. This study aimed to evaluate global practice patterns among surgeons affiliated with the International Federation for the Surgery and Other Therapies for Obesity (IFSO), identifying key variations and factors influencing intraoperative decision-making.

**Methods:**

A cross-sectional survey was distributed between January and November 2025 to active IFSO members performing RYGB. The survey explored techniques related to the gastric pouch configuration, intestinal limb lengths, gastrojejunostomy, ring augmentation, and adjunct use. Responses from 245 surgeons across 47 countries were analyzed using descriptive statistics, subgroup comparisons, and multivariable logistic regression.

**Results:**

Technical variability was widespread. Only 53.5% routinely measured gastric pouch length; 67.3% described their pouches as “short” or “small,” with variable anatomical endpoints. Bougie sizes ranged from 28 to 46 Fr, with 36 Fr being most common (41.2%). Only 21.6% of respondents reported routinely measuring total bowel length. Ring-augmentation was used by 20.4%, with significant regional and experiential variation. Linear stapling was preferred for gastrojejunostomy (81.2%), but stoma size and suture materials varied. Routine closure of both Petersen’s space and the jejunojejunal mesenteric defect was common (69%) yet non-uniform. High-volume surgeons had shorter hospital stays and longer follow-up, but technical patterns were similar.

**Conclusion:**

Global RYGB practice exhibits heterogeneity in several key operative steps. Improved reporting of intraoperative technical variables may help clarify which differences are most relevant to perioperative, long-term, and registry-based outcomes, and may support future consensus-building in areas where standardization is clinically justified.

## Key points

A global survey of 245 metabolic and bariatric surgeons across 47 countries revealed significant variation in RYGB techniques, including gastric pouch size, limb lengths, and anastomotic approaches.

Only 53.5% of surgeons routinely measured gastric pouch length, and most relied on subjective descriptors, highlighting a lack of standardization in pouch configuration.

Fixed intestinal limb lengths were used by 93% of respondents (median BPL: 100 cm; AL: 120 cm), while only 21.6% measured total bowel length, underscoring limited tailoring to patient anatomy.

Ring augmentation was used by a minority of respondents (20.4%), with use more closely associated with surgeon experience than with annual case volume.

High-volume surgeons demonstrated shorter hospital stays and longer follow-up but did not show greater technical consistency, indicating that experience alone does not ensure procedural standardization.

## Introduction

Obesity is a chronic disease marked by adipose tissue dysfunction and metabolic disruption [1]. According to the 2025 Lancet Commission, over 1 billion people worldwide are affected, leading to increased cases of type 2 diabetes, cardiovascular disease, and economic costs projected to exceed $4.3 trillion annually by 2035, especially in low- and middle-income countries [1, 2]. In response, the 2022 ASMBS/IFSO guidelines highlight metabolic and bariatric surgery (MBS) as the most effective long-term treatment for obesity and its related diseases [3]. Together, both the ASMBS/IFSO guidelines and the 2025 Lancet Commission broadened surgical eligibility beyond BMI thresholds alone and emphasize early interventional considerations for patients with metabolic risk, even at lower BMI ranges [4].

Among the various surgical interventions available, sleeve gastrectomy (SG) has emerged as the most common surgical intervention globally due to its ease and positive outcomes [2, 5, 6]. Roux-en-Y gastric bypass (RYGB), although less frequently performed in some areas [2, 6], remains the second most common procedure, noted for its effectiveness in weight loss and quality of life improvements [5, 7, 8]. Developed in the 1960s and refined in the 1990s, RYGB facilitates both restrictive and hypoabsorptive effects, producing reliable long-term results and better management of conditions like gastroesophageal reflux disease (GERD) [9–13].

However, RYGB techniques vary significantly around the world, influenced by differing surgeon practices concerning gastric pouch size, intestinal limb lengths, and gastrojejunostomy construction [14–17]. Areas of disagreement include closure of Petersen’s space and the jejunojejunal mesenteric defect, as well as the use of ring-augmentation, driven by variations in regional training and experience [14, 18]. Despite some connections between limb length configurations and metabolic outcomes, individual differences in small bowel length complicate standardized protocols [16, 19]. The use of Esophagogastroduodenoscopy (EGD) pre- and post-operatively is similarly shaped by personal preferences, training backgrounds, and economic factors [2, 14, 20].

This inconsistency in RYGB techniques may impact different outcome domains in different ways, with some technical variables being more relevant to perioperative safety and complication profiles, and others more closely related to long-term weight-loss, metabolic, or nutritional outcomes, underscoring a need for deeper insights into current practices. This study aims to assess global RYGB techniques among the International Federation for the Surgery and Other Therapies for Obesity (IFSO) members and establish a foundation for future evidence-based consensus in surgical education and practices.

## Methods

### Study Design

This cross-sectional international survey aimed to evaluate global technical variations in the performance of primary RYGB among MBS surgeons affiliated with IFSO. Developed in English, the survey was distributed via Google Forms from January to November 2025, adhering to the STROCSS criteria [21].

### Survey Content

The survey explored various intraoperative decision points, including the use and application of ring-augmentation, criteria for its use, and ring diameter. It also examined gastric pouch creation practices, such as gastric pouch size measurement, objective and subjective estimation methods, anatomical termination points, bougie sizes, and reinforcement techniques. Respondents were questioned about small bowel configuration, including total bowel length measurement, the determination of biliopancreatic and alimentary limb lengths, and the resection or reinforcement of the gastric remnant (Table 1). In the survey, the term ‘middle gastric vein’ was used to refer to the second short gastric vessel encountered during pouch construction.

**Table 1:** Survey instrument evaluating technical variations in primary Roux-en-Y gastric bypass (RYGB) among IFSO-affiliated metabolic and bariatric surgery (MBS) surgeons

Question	Answer Choices
Ringed vs. Non-Ringed RYGB	
A) Do you routinely use a ring for your Primary RYGB procedures?	Yes
	No
	According to the Patient
B) If you use a Ring, which application technique do you prefer?	Pars Flaccida
	Peri-gastric Technique
C) what diameter of ring do you typically use?	*Open Question*
D) If you apply it according to the Patient, which patients do you prefer to apply the ring to? (If you routinely apply Ring in primary procedures, please do not answer this question) *(Can choose more than one answer)*	Male Sex
	Female Sex
	BMI > 40 kg/m 2
	BMI > 45 kg/m2
	BMI > 50 kg/m2
	BMI > 55 kg/m2
*Gastric Pouch*	
A) Do you regularly measure the size of the gastric pouch in (cm)?	Yes
	No
B) If yes, what is the typical size of the gastric pouch you create in (cm)?	*Open Question*
C) How would you describe the size of the gastric pouch you perform, subjectively	Micro Pouch
	Short or Small pouch
	Long pouch
D) At what level does your gastric pouch end?	Between the hiatus and the first gastric vein
	Between the first and the middle gastric vein (2nd gastric vein)
	Just below the middle gastric vein (2nd gastric vein)
	Between option c and the incisura
E) Do you routinely reinforce the gastric pouch?	Yes
	No
F) If yes, what method of reinforcement do you prefer?	Barbed sutures
	Non-barbed sutures
	Clips
	Other
G) What is the Bougie Size you use in FR?	*Open Question*
Do you routinely count all the bowel length in Primary RYGB? (Yes / No)	
Do You perform your anastomosis at a certain percentage of the small bowel length in Primary RYGB (Yes / No)	
If yes, add the percentage (*Open Question)*	
*Bilioenteric Limb*	
What is the typical length of the bilioenteric limb you create in (cm) from the DJ in Primary RYGB?	*Open Question*
*Alimentary Limb*	
What is the typical length of the Alimentary limb you create in (cm) from the GJ in Primary RYGB?	*Open Question*
*Common Channel*	
What is the typical length of the common channel you create in (cm)?	*Open Question*
	I do not count the Common channel
*Gastric Remnant*	
A) Do you routinely resect the gastric remnant?	Yes
	No
B) If yes, why?	*Open Question*
C) If No, do you reinforce the gastric remnant?	Yes
	No
D) If yes, what method of reinforcement do you prefer?	Barbed sutures
	Non-barbed sutures
	Clips
	Other
*Pre- and Post-operative Endoscopy*	
A) Do you routinely perform pre-operative endoscopy before Primary RYGB?	Yes
	No
B) Do you routinely perform post-operative endoscopy after Primary RYGB?	Yes
	No
*Gastrojejunostomy Stoma*	
A) What is the typical diameter of the gastrojejunostomy stoma you create in (cm)?	*Open Question*
B) What type of stapler do you typically use for the gastrojejunostomy?	Linear
	Circular
	Hand-sewn
C) If linear stapler, what size of reload do you typically use in (mm)?	*Open Question*
D) If circular stapler, what size of reload do you typically use?	*Open Question*
E) If hand-sewn, what type of sutures do you typically use?	Barbed
	Non-barbed
*Defect Closure*	
A) Do you routinely close all defects (Peatreson’s space, mesenteric defect, etc.)?	Yes
	No
	Not all defects
B) If not all defects, which defects do you typically close? *(Can choose more than one answer)*	Peatreson’s space
	jejunojejunal mesenteric defect
C) What type of closure do you use to close the defects	Barbed Sutures
	Non-Barbed Sutures
	Clips
	Other
*Drainage*	
A) Do you routinely insert a drain after Primary RYGB?	Yes
	No
	On patient selection
B) If on Patient Selection Which patients do you place a drain in Primary RYGB?	*Open Question*
*Leak Tests*	
A) Do you routinely perform an intraoperative leak test in Primary RYGB?	Yes
	No
	On patient selection
B) Do you routinely perform an Intraoperative endoscopic leak test?	Yes
	No
	On patient selection
*Indocyanine Green*	
Do you routinely use indocyanine green during RYGB?	Yes
	No
	On patient selection
*Postoperative Care*	
A) What is the length of the typical hospital stay after RYGB for most of your patients in Days?	*Open Question*
B) What is the typical follow-up period after RYGB in Months?	*Open Question*
*Postoperative Imaging*	
A) Do you routinely order a postoperative CT contrast study?	Yes
	No
B) Do you routinely order a postoperative barium X-ray?	Yes
	No
C) When do you perform post-operative imaging?	Immediately post-operative
	At time of discharge
	One week Follow up
	One month follow up
	Option b plus c
	Option b plus d
	On patient basis
*Open-Ended Questions*	
1) Do you have a preferred technique for performing RYGB? (Yes / No) (If yes, please explain the technique briefly)	
2) Would you consider changing your preferred technique in the future? ( If yes, please explain your reason.)	
3) What are the most common complications associated with RYGB in your experience?	
4) Are there any emerging techniques or technologies that you believe could improve RYGB outcomes?	
5) What are the biggest challenges you face when performing RYGB?	

*RYGB* Roux-en-Y gastric bypass, *IFSO* International Federation for the Surgery of Obesity and Metabolic Disorders, *MBS* metabolic and bariatric surgery *Fr* French gauge, *DJ* duodenojejunal flexure, *GJ* gastrojejunostomy, *ICG* indocyanine green, *CT* computed tomography

The survey delved into gastrojejunostomy techniques, the preferred anastomotic methods (linear stapled, circular stapled, or hand-sewn), stoma diameter, stapler reload sizes, and adherence to standard techniques. It also covered the management of the Petersen’s space and the jejunojejunal mesenteric defect, including routine closure techniques. Furthermore, it assessed the use of intraoperative adjuncts like leak testing (methylene blue, air insufflation, or endoscopy), indocyanine green imaging, and drain placement. Postoperative protocols, typical hospital stay lengths, and follow-up care structure were also included, along with open-ended questions about technical challenges and views on emerging technologies to enhance surgical outcomes (Table 1).

### Survey Validity Assessment

A structured content validity evaluation was conducted before distributing the survey to ensure the relevance of items. Ten expert MBS surgeons reviewed 52 items, rating each on a four-point Likert scale (1 = not relevant, 4 = highly relevant). The item-level content validity index (I-CVI) was calculated by dividing the number of experts rating an item as 3 or 4 by the total number of experts. Overall scale validity was assessed using the average scale-level content validity index (S-CVI/Ave), which was 0.857, and the universal agreement scale-level index (S-CVI/UA), at 0.788, indicating strong validity and substantial agreement among experts.

### Data Collection and Survey Distribution

To coordinate survey development and dissemination, a dedicated research group (the TRPL–IFSO Task Force) was established through The Research Papyrus Lab (TRPL), a collaborative academic initiative committed to supporting global research. Members of the TRPL-IFSO Task Force were responsible for constructing a comprehensive contact database by systematically reviewing the IFSO membership directory available on the organization’s official website (https://www.ifso.com/find-a-member/) [22]. When direct email details were not available through the directory, additional publicly accessible professional sources (e.g., institutional pages and professional profiles) were used to identify contact information. Only surgeons verified as IFSO members and actively performing MBS were retained in the outreach list.

The English-language survey was hosted on Google Forms and distributed to IFSO-affiliated MBS surgeons in two waves during 2025. The initial invitation was circulated in January 2025, with reminders in February and March. The survey was initially closed on March 15, 2025. To improve regional representativeness, the survey was reopened in September 2025 and remained open until November 2025. During the second wave, TROGSS - The Robotic Global Surgical Society supported dissemination by distributing survey invitations to IFSO-member surgeons within its database; TROGSS had no role in data access, data management, or analysis. All obtained contacts were organized by region of practice and surgical profile to facilitate targeted reminders. Invitations were emailed to 1,043 surgeons with available contact details; 530 emails were returned as undeliverable (bounce-backs), leaving 513 delivered invitations. We received 252 submissions (49.1% of delivered), and 245 eligible complete responses were included in the final analysis (47.8% of delivered) after excluding incomplete submissions.

Eligibility required confirmation of IFSO membership, current performance of MBS as primary operator, and electronic informed consent for anonymized data use. Participants were also offered the option to have their names and affiliations listed as collaborators; those who declined were not listed.

### Subdivision by IFSO Regional Chapters

To enable regional comparisons, participants were categorized according to the five official IFSO chapters based on their country of practice. These chapters included: the North American Chapter (NAC), Latin American Chapter (LAC), European Chapter (EC), Middle East and North Africa Chapter (MENAC), and Asia-Pacific Chapter (APC), in line with classifications published on the IFSO website.

### Surgeon Characteristics and Classification of Surgical Caseload

Collected variables included the surgeon’s country of practice, years of MBS experience, and annual volume of primary RYGB procedures. Surgeons performing ≥ 100 MBS procedures annually were categorized as high-volume surgeons. Frequency of assisting or observing RYGB was also recorded to assess institutional exposure.

### Authorship Criteria and Attribution

Authorship for this study followed the criteria set by the International Committee of Medical Journal Editors (ICMJE). Contributors were divided into collaborative consortia or designated as main authors based on their involvement. The IFSO RYGB GLOBAL consortium included all MBS surgeons who participated in the survey and consented to their inclusion. This group also featured an expert panel of surgeons who assessed the survey’s content validity, highlighting their contributions to the study. Additionally, the TRPL-IFSO Task Force Consortium was formed to collect and verify contact information for eligible surgeons. Although they did not participate in the survey or data analysis, their efforts in building a comprehensive participant database were essential. Main authorship was given to those who significantly contributed to the research and manuscript development, including study design, content validation, data analysis, and manuscript preparation. The main authors met ICMJE criteria and approved the final manuscript before submission.

### Statistical Analysis

Statistical analysis was conducted using SPSS version 25 (IBM Corp.). Categorical variables are summarized as frequencies and percentages, and continuous variables as medians with interquartile ranges (IQR). The Shapiro-Wilk test was used to assess the normality of continuous data. For between-chapter comparisons, categorical variables, including dichotomous variables compared across more than two groups, were analyzed using Pearson’s χ² test or Fisher’s exact test, as appropriate, whereas continuous variables were compared using the Kruskal–Wallis test. For volume-based comparisons between two groups, categorical variables were analyzed using Pearson’s χ² test when all expected cell counts were ≥ 5 or Fisher’s exact test was used when any expected cell count was < 5, and continuous variables using the Mann–Whitney U test. A multivariable logistic regression model was used to identify factors associated with ring use in primary RYGB. Correlations between continuous or ordinal variables were assessed using Spearman’s rank correlation, and a two-sided p-value < 0.05 was considered statistically significant.

To evaluate variation by surgical volume, a subgroup analysis compared low-volume (< 100 MBS procedures annually) and high-volume (≥ 100 annually) surgeons, using 1:1 propensity score matching based on IFSO chapter, years of experience, and procedure type. This resulted in two matched cohorts of 60 surgeons each for volume-based comparisons within each technical domain. Moreover, a post hoc power analysis was conducted using G*Power (version 3.1.9.7) for chi-square tests of independence to assess the achieved statistical power for detecting regional variations across selected key technical practices. Effect sizes were estimated using Cohen’s w based on observed distributions across the five IFSO regions. Analyses were performed with an alpha level of 0.05, using the corresponding degrees of freedom for each outcome and a total sample size of 245 participants.

## Results

### Participant Characteristics and Experience

**Fig. 1:**
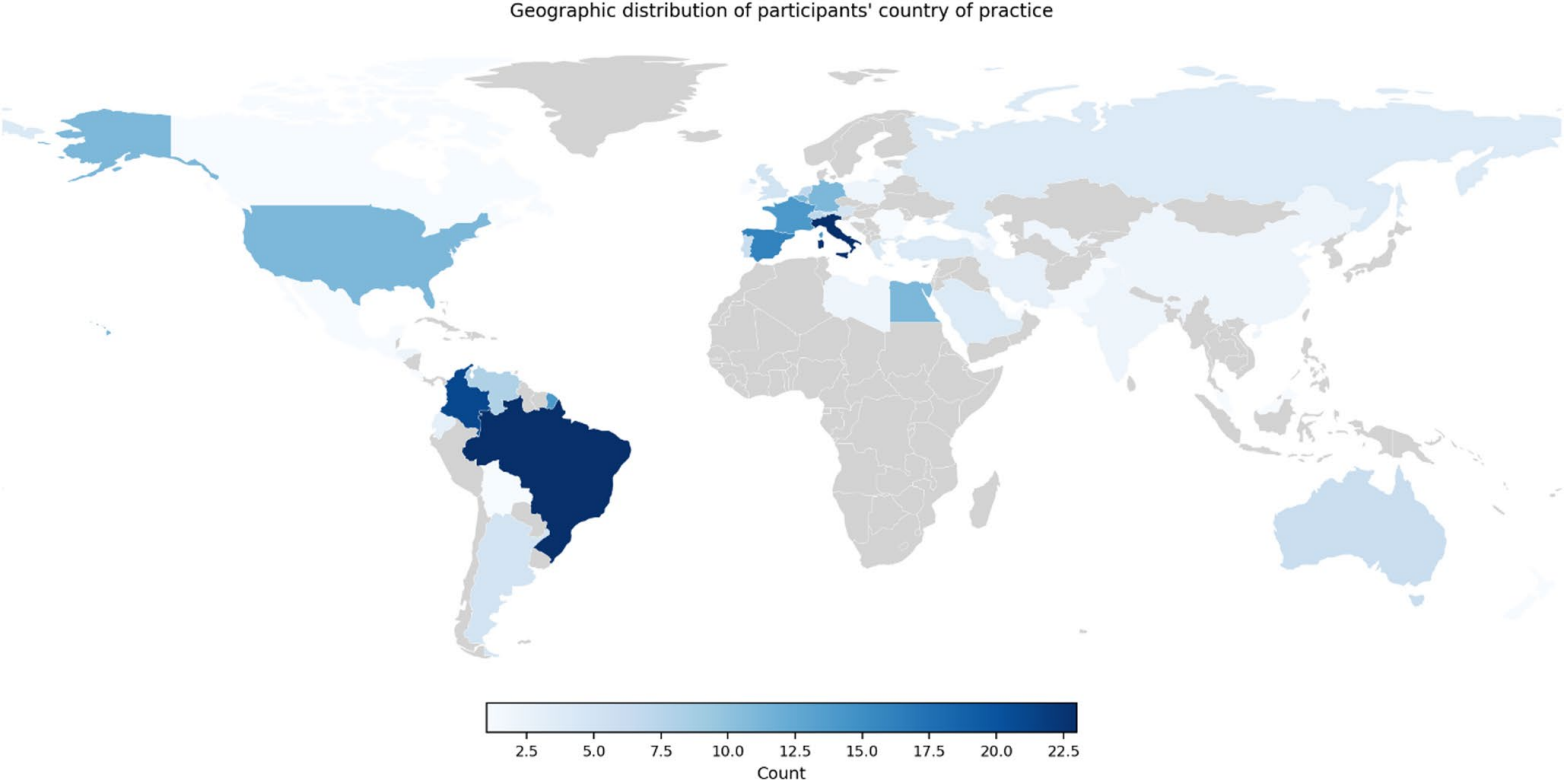
World choropleth showing the number of participating IFSO-affiliated metabolic and bariatric surgeons by country of practice. Shading intensity increases with higher respondent counts (light = fewer, dark = more); countries with no respondents are shown in grey

**Fig. 2:**
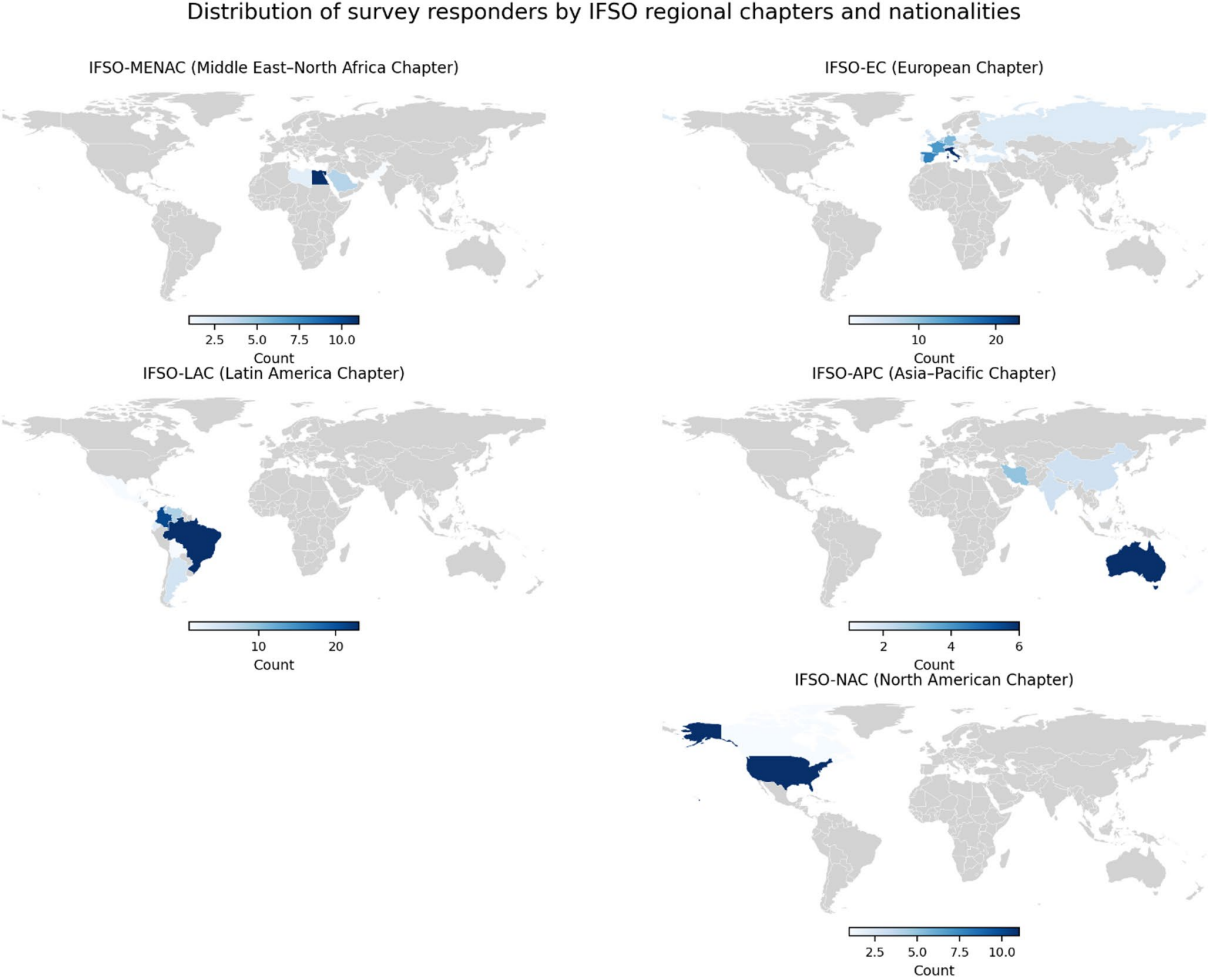
Panel choropleths stratified by IFSO regional chapter (IFSO-MENAC, IFSO-EC, IFSO-LAC, IFSO-APC, IFSO-NAC) showing respondent counts by country of practice within each chapter. Shading intensity increases with higher counts (light = fewer, dark = more); countries with no respondents are shown in grey. Overseas territories embedded in base map polygons were not displayed as separate regions for clarity

Out of 252 responses, 245 were eligible for analysis after excluding six incomplete responses. Participants represented 47 countries across all five IFSO regional chapters (Fig. 1). 50.2% were affiliated with the EC, followed by LAC (17.6%), MENAC (12.2%), APC (10.6%), and NAC (9.4%) (Fig. 2) (Table 2). The median MBS experience was 14 years (IQR: 8–20). On average, respondents performed 50 primary RYGB surgeries annually as lead surgeons (IQR: 25–100) and attended 75 primary RYGB procedures annually in any role (IQR: 32.5–125) (Table 3).

**Table 2:** Distribution of survey respondents by IFSO regional chapter (total *n* = 245)

Demographic data	*N*(%)
Latin America Chapter	43 (17.6)
Middle East-North Africa Chapter	30 (12.2)
Asia-Pacific Chapter	26 (10.6)
European Chapter	123 (50.2)
North American Chapter	23 (9.4)

*APC* Asia–Pacific Chapter, *EC *European Chapter, *LAC* Latin America Chapter, *MENAC* Middle East–North Africa Chapter, *NAC* North American Chapter. Data are n (%)

**Table 3:** Surgical experience and operative exposure among survey respondents

	*n* (%) median (Q1 - Q3)
Years of Experience in Metabolic and Bariatric Surgery	14 (8–20)
Annual Volume of Primary RYGB Procedures as a primary surgeon	50 (25–100)
The Annual Number of Primary RYGB Procedures attended generally	75 (32.5–125)
Preferred technique for performing RYGB	175 (71.4)

Data are presented as median (Q1–Q3) for continuous variables and n (%) for categorical variables. Q1–Q3, first–third quartile; RYGB, Roux-en-Y gastric bypass. n = number of surgeons reporting a preferred technique for the corresponding variable

### Gastric Pouch Configuration

A total of 131 surgeons (53.5%) routinely measured the gastric pouch during primary RYGB, with a median reported length of 6 cm (IQR: 5–7 cm) (Table 4). Overall, 165 respondents (67.3%) described their pouch as short or small, 30.6% as long, and 2.0% as a micro pouch (Table 4). The most commonly reported termination level was between the first and middle gastric vein (2nd gastric vein) (53.9%), followed by just below the middle gastric vein (27.3%) (Table 4). No significant volume-based differences were identified in pouch measurement or termination level (Table 5). Routine reinforcement of the gastric pouch was reported by 56 surgeons (22.8%) (Table 4). Among these, non-barbed sutures were the most commonly used method (50.0%), followed by clips or other materials (26.8%) and barbed sutures (23.2%) (Table 4). Reinforcement methods varied significantly across IFSO chapters (*p* = 0.002) (Table 4). In contrast, reinforcement rates and material choice did not differ significantly by surgical volume (30.0% in low-volume vs. 25.0% in high-volume surgeons, *p* = 0.596) (Table 5). 

**Table 4:** Technical aspects of application of rings, gastric pouch and anastomosis and limbs in the primary RYGB as reported by the participants of the survey, stratified by IFSO regional chapter

	Total *n*= 245	Latin America *n*= 43	Middle East-North Africa *n*= 30	Asia-Pacific *n*= 26	European *n*= 123	North American *n*= 23	*P*-value
Use of ring-augmentation for Primary RYGB procedures routinely	50 (20.4)	5 (11.6)	5 (16.7)	8 (30.8)	32 (26)	0 (0)	0.092
- Peri-gastric Technique	37 (74)	3 (60)	1 (20)	7 (87.5)	26 (81.3)	0 (0)	**0.021**
- Pars Flaccida	13 (26)	2 (40)	4 (80)	1 (12.5)	6 (18.8)	0 (0)	
Diameter of the ring	7 (6.5 - 7.5)	7 (7 - 7)	7.25 (6.6 - 7.5)	7 (6.4 - 7.5)	7 (7 - 7.5)	0	**0.084**
Regularly measure the size of the gastric pouch (cm)	131 (53.5)	23 (53.5)	20 (66.7)	12 (46.2)	61 (49.6)	15 (65.2)	0.322
Typical size of the gastric pouch	6 (5 - 7)	5 (4 - 6)	6 (5 - 6)	7 (6 - 8)	6 (5 - 7)	5 (5 - 7)	**0.034**
Description of size of the gastric pouch							0.300
- Long pouch	75 (30.6)	6 (14)	11 (36.7)	10 (38.5)	42 (34.1)	7 (30.4)	
- Short or Small pouch	165 (67.3)	36 (83.7)	19 (63.3)	15 (57.7)	79 (64.2)	16 (69.6)	
- Micro Pouch	5 (2)	1 (2.3)	0 (0)	1 (3.8)	2 (1.6)	0 (0)	
Level of gastric pouch end							**0.047**
- Between the hiatus and the first gastric	19 (7.8)	3 (7)	2 (6.7)	3 (11.5)	8 (6.5)	3 (13)	
- Between the first and the middle gastric vein (2^nd^ gastric vein)	132 (53.9)	29 (67.4)	12 (40)	12 (46.2)	69 (56.1)	10 (43.5)	
- Just below the middle gastric vein	67 (27.3)	9 (20.9)	15 (50)	5 (19.2)	33 (26.8)	5 (21.7)	
- Between the middle gastric vein and the incisura	27 (11)	2 (4.7)	1 (3.3)	6 (23.1)	13 (10.6)	5 (21.7)	
Reinforce the gastric pouch routinely	56 (22.8)	18 (41.9)	11 (36.7)	6 (23.1)	17 (13.8)	5 (21.7)	**0.002**
Barbed sutures	13 (23.2)	3 (16.7)	3 (27.3)	3 (50)	3 (17.6)	1 (20)	
Clips	10 (17.9)	0 (0)	4 (36.4)	1 (16.7)	5 (29.4)	0 (0)	
Non-barbed sutures	28 (50)	15 (83.3)	4 (13.3)	1 (16.7)	5 (29.4)	4 (80)	
Other	5 (8.9)	0 (0)	0 (0)	1 (16.7)	4 (23.5)	0 (0)	
Bougie Size use in FR							**0.006**
28	4 (1.6)	0 (0)	0 (0)	0 (0)	4 (3.3)	0 (0)	
32	33 (13.5)	16 (37.2)	3 (10)	1 (3.8)	9 (7.3)	4 (17.4)	
33	2 (1)	1 (2.3)	0 (0)	0 (0)	1 (0.8)	0 (0)	
34	25 (10.2)	3 (7)	1 (3.3)	2 (7.7)	14 (11.4)	5 (21.7)	
35	4 (1.6)	0 (0)	0 (0)	0 (0)	4 (3.3)	0 (0)	
36	101 (41.2)	15 (34.9)	13 (43.3)	16 (61.5)	51 (41.5)	6 (26.1)	
37	2 (0.8)	0 (0)	0 (0)	0 (0)	1 (0.8)	1 (4.3)	
38	24 (9.8)	1 (2.3)	5 (16.7)	3 (11.5)	15 (12.2)	0 (0)	
39	3 (1.2)	0 (0)	0 (0)	0 (0)	3 (2.4)	0 (0)	
40	26 (10.6)	3 (7)	6 (20)	3 (11.5)	11 (8.9)	3 (13)	
41	1 (0.4)	0 (0)	0 (0)	0 (0)	1 (0.8)	0 (0)	
42	6 (2.4)	1 (2.3)	1 (3.3)	0 (0)	3 (2.4)	1 (4.3)	
46	1 (0.4)	0 (0)	0 (0)	1 (3.8)	0 (0)	0 (0)	
Routinely count all the bowel length in Primary RYGB	56 (22.9)	6 (14)	7 (23.3)	10 (38.5)	26 (21.1)	7 (30.4)	0.166
Typical length of the bilioenteric limb created in (cm) from the DJ in Primary RYGB	100 (77.5 - 120)	100 (100 - 130)	100 (100 - 131.25)	100 (70 - 120)	100 (70 - 120)	100 (75 - 120)	0.103
Typical length of the Alimentary limb created in (cm) from the GJ in Primary RYGB	120 (100 - 150)	100 (100 - 150)	100 (100 - 125)	100 (78.8 - 105)	135 (100 - 150)	150 (100 - 150)	**<0.001**
Typical length of the common channel created in (cm)	300 (175 - 325)	120 (120 - 120)	350 (250 - 350)	200 (200 - 200)	300 (187.5 - 300)	-	0.220

Data are *n* (%) unless otherwise indicated; continuous variables are median (Q1–Q3). Ring diameter and gastrojejunostomy stoma diameter are reported in cm; limb lengths are reported in cm (biliopancreatic limb from DJ, alimentary limb from GJ, and common channel); bougie size is reported in French gauge (Fr). *P*-values for between-chapter comparisons were derived using Pearson’s χ² test for categorical variables when all expected cell counts were ≥5 or Fisher’s exact test when any expected cell count was <5, and the Kruskal–Wallis test for continuous variables (two-sided). GJ: gastrojejunostomy

**Table 5:** Volume-based comparison of technical practices in primary RYGB (low- vs. high-volume surgeons)

	**Low volume** ***n*** **= 60**	**High volume** ***n*** **= 60**	***P*** **-value**
Use a ring for Primary RYGB procedures routinely	17 (28.3)	19 (31.7)	0.543
- Peri-gastric Technique	12 (70.6)	16 (80)	0.703
- Pars Flaccida	5 (29.4)	4 (20)	
Diameter of ring	7 (6.5 - 7.5)	7 (7 - 7.5)	0.211
Regularly measure the size of the gastric pouch	30 (50)	32 (53.3)	0.855
Typical size of the gastric pouch	6 (5 - 6.5)	6 (4 - 7)	0.906
Description of size of the gastric pouch			0.094
- Long pouch	14 (23.3)	23 (38.3)	
- Short or Small pouch	45 (75)	34 (56.7)	
- Micro Pouch	1 (1.7)	3 (5)	
Level of gastric pouch end			0.210
- Between the hiatus and the first gastric	6 (10)	2 (3.3)	
- Between the first and the middle gastric vein	30 (50)	37 (61.7)	
- Just below the middle gastric vein (2^nd^ gastric vein)	18 (30)	12 (20)	
- Between below the middle gastric vein and the incisura	6 (10)	9 (15)	
Reinforce the gastric pouch routinely	18 (30)	15 (25)	0.596
Barbed sutures	4 (22.2)	4 (26.7)	
Clips	4 (22.2)	3 (20)	
Non-barbed sutures	8 (44.4)	8 (53.3)	
Other	2 (11.1)	0 (0)	
Bougie Size use in FR			0.207
28	1 (1.7)	1 (1.7)	
32	9 (15)	7 (11.7)	
33	1 (1.7)	0 (0)	
34	3 (5)	10 (16.7)	
35	0 (0)	2 (3.3)	
36	25 (41.7)	24 (40)	
37	1 (1.7)	0 (0)	
38	5 (8.3)	5 (8.3)	
39	0 (0)	0 (0)	
40	5 (8.3)	7 (11.7)	
41	0 (0)	1 (1.7)	
42	4 (6.7)	0 (0)	
46	0 (0)	0 (0)	
Routinely count all the bowel length in Primary RYGB	18 (30)	10 (16.7)	0.130
Typical length of the bilioenteric limb created in (cm) from the DJ in Primary RYGB	100 (80 - 120)	100 (75 - 120)	0.957
Typical length of the Alimentary limb created in (cm) from the GJ in Primary RYGB	120 (100 - 150)	120 (100 - 150)	0.839
Typical length of the common channel created in (cm)	300 (300 - 375)	200 (200 - 200)	0.333
Routinely resect the gastric remnant	0 (0)	1 (1.7)	1.000
Reinforce the gastric remnant	19 (31.7)	16 (26.7)	0.968
Barbed sutures	4 (21.1)	4 (25)	
Clips	8 (42.1)	7 (43.8)	
Non-barbed sutures Other	5 (26.3) 2 (10.5)	4 (25) 1 (6.3)	
Routinely perform pre-operative endoscopy before Primary RYGB	53 (88.3)	45 (75)	0.097
Routinely perform post-operative endoscopy after Primary RYGB	14 (23.3)	10 (16.7)	0.494
Typical diameter of the gastrojejunostomy stoma (cm)	3 (2 - 3.5)	2.5 (2 - 3.4)	0.204
Type of stapler typically used for the gastrojejunostomy			0.168
Circular	2 (3.3)	4 (6.7)	
Hand-sewn	5 (8.3)	11 (18.3)	
Linear	53 (88.3)	45 (75)	
Size of reload used in linear stapler (mm)	45 (35.7 - 45)	45 (31.3 - 48.8)	0.873
Size of reload used in circular stapler (mm)	23 (21 - 23)	25 (22 - 25)	1.000
Sutures used in hand-sewn anastomosis			0.308
Barbed	4 (80)	5 (45.5)	
Non-barbed	1 (20)	6 (54.5)	
Routinely close all defects (Petersen’s space, jejunojejunal mesenteric defect, etc.)			0.261
Yes, all defects	38 (63.3)	46 (76.7)	
Yes, not all defects	16 (26.7)	11 (18.3)	
Jejunojejunal Mesenteric defect	12 (66.7)	8 (72.7)	
Petersen’s space	6 (33.3)	3 (27.3)	
No	6 (10)	3 (5)	
Type of closure used to close the defects			0.129
Barbed Sutures	29 (48.3)	22 (38.6)	
Non- Barbed Sutures	21 (35)	25 (43.9)	
Clips or Endo hernia Staplers	2 (3.3)	8 (13.3)	
Surgical Glue	1 (1.7)	3 (5)	
Routinely insert a drain after Primary RYGB			0.889
Yes	14 (23.3)	15 (25)	
On patient selection	3 (5)	2 (3.3)	
No	43 (71.7)	43 (71.7)	
Routinely perform an intraoperative leak test in Primary RYGB	55 (91.7)	51 (85)	0.394
Routinely perform an Intraoperative endoscopic leak test	10 (16.7)	8 (13.3)	0.799
Routinely use indocyanine green (ICG) during RYGB	6 (10)	6 (10)	1.000
Typical length of typical hospital stays after RYGB in Days	2 (1 - 3)	1 (1 - 2)	**0.002**
Typical follow-up period after RYGB in Months	12 (3 - 24)	21 (12 - 60)	**0.042**
Routinely order a postoperative CT contrast study	5 (8.3)	1 (1.7)	0.207
Routinely order a postoperative barium X-ray	14 (23.3)	2 (3.3)	**0.002**

High-volume defined as ≥100 primary RYGB procedures/year; low-volume defined as <100 procedures/year. Data are *n* (%) unless otherwise indicated; continuous variables are median (Q1–Q3). *P*-values reflect between-group comparisons using χ²/Fisher’s exact tests for categorical variables and Mann–Whitney U tests for continuous variables (two-sided). Fr, French gauge; DJ, duodenojejunal flexure; GJ, gastrojejunostomy

### Bougie/Calibration Tube

Bougie or calibration tube size during primary RYGB ranged from 28 to 46 Fr, with 36 Fr being the most commonly used size overall, reported by 101 surgeons (41.2%) (Table 4). Other frequently used sizes included 32 Fr (13.5%), 34 Fr (10.2%), 38 Fr (9.8%), and 40 Fr (10.6%) (Table 4). Bougie size preferences differed significantly across IFSO chapters (*p* = 0.006) (Table 4). No significant difference in bougie size distribution was observed between low-volume and high-volume surgeons (*p* = 0.207), with 36 Fr remaining the most common size in both groups (41.7% vs. 40.0%, respectively) (Table 5). Among ring users, bougie size was not correlated with ring diameter (*r* = 0.019, *p* = 0.904) (Table 6).

**Table 6:** Associations between surgeon volume and ring augmentation practices in primary RYGB

	Variable	median (Q1 - Q3)	aOR (95% CI) or *r*	*P*-value
Annual number of primary RYGB procedures attended, stratified by ring technique among ring users	Peri-gastric Technique	80 (45–200)		0.635
	Pars Flaccida	65 (33–157)		
Correlation Analysis	**Bougie Size use in FR vs. Diameter of ring**		0.019	0.904
Multivariable Logistic Regression Analysis of Factors Associated with Routine Ring Use	**Annual volume (Primary surgeon)**		aOR = 1.000 (95% CI: 0.999–1.001)	0.870
	**Annual RYGB cases attended (general)**		aOR = 1.001 (95% CI: 1.000–1.002)	0.231
	**Years of Experience** **(Bariatric surgery)**		**aOR = 1.049 ** **(95% CI: 1.005–1.096)**	**0.030**

Continuous data are median (Q1–Q3). r denotes correlation coefficient (Spearman). aOR denotes adjusted odds ratio from multivariable logistic regression with 95% confidence interval (CI); outcome = routine ring use. P-values are two-sided

### Use of Ring Augmentation

Of the 245 respondents, 50 surgeons (20.4%) reported using ring augmentation during primary RYGB (Table 4). Among ring users, the peri-gastric technique was more common than the pars flaccida approach (74.0% vs. 26.0%), and the preferred technique differed significantly across IFSO chapters (*p* = 0.021) (Table 4). No respondents in the NAC reported using ring augmentation (Table 4). Among surgeons who used a ring, the median reported ring diameter was 7.0 cm (IQR: 6.5–7.5), with no significant difference across IFSO chapters (*p* = 0.084) (Table 4). In volume-based subgroup analysis, ring use did not differ significantly between low-volume and high-volume surgeons (28.3% vs. 31.7%, *p* = 0.543), and ring technique and diameter were also comparable between groups (Table 5). Correlation analysis showed no association between annual RYGB caseload and ring use (*p* = 0.635), and no correlation between bougie size and ring diameter among ring users (*r* = 0.019, *p* = 0.904) (Table 6). In multivariable logistic regression, years of experience in metabolic and bariatric surgery were independently associated with ring use (aOR 1.049, 95% CI: 1.005–1.096; *p* = 0.030), whereas annual RYGB case volume as primary surgeon (*p* = 0.870) and the annual number of procedures attended overall (*p* = 0.231) were not significantly associated with ring use (Table 6).

### Gastrojejunostomy Construction

The median diameter of the gastrojejunostomy (GJ) stoma during primary RYGB was 3.0 cm (IQR: 2.0–3.75), with significant variation across IFSO chapters (*p* = 0.024) (Table 7). No significant difference in stoma diameter was observed between low- and high-volume surgeons (*p* = 0.204) (Table 5). Linear stapling was the predominant GJ construction technique, used by 199 surgeons (81.2%), followed by hand-sewn anastomosis in 36 (14.7%) and circular stapling in 10 (4.1%) (Table 7). Preferred GJ construction technique differed significantly across IFSO chapters (*p* = 0.001) (Table 7), whereas no significant difference was observed between low- and high-volume surgeons (*p* = 0.168) (Table 5). Among surgeons using linear staplers, the median reload size was 45 mm (IQR: 34.25–45), with no significant difference by surgical volume (*p* = 0.873) (Table 5) (Table 7). Among hand-sewn anastomoses, barbed sutures were used slightly more often than non-barbed sutures (53.8% vs. 46.2%), with no significant difference between volume groups (*p* = 0.308) (Table 5) (Table 7).

**Table 7:** Technical practices related to gastric remnant management, endoscopy use, gastrojejunostomy construction, and mesenteric defect closure in primary RYGB, stratified by IFSO regional chapter

	Total *n* = 245	Latin America *n* = 43	Middle East-North Africa *n* = 30	Asia-Pacific *n* = 26	European *n* = 123	North American *n* = 23	*P*-value
Routinely resect the gastric remnant	1 (0.4)	0 (0)	0 (0)	0 (0)	1 (0.8)	0 (0)	0.910
Reinforce the gastric remnant Barbed sutures Clips Non-barbed sutures Other	54 (22) 10 (18.5) 24 (44.4) 14 (25.9) 6 (11.1)	12 (27.9) 1 (8.3) 0 (0) 11 (91.7) 0 (0)	12 (40) 2 (16.7) 8 (66.7) 2 (16.7) 0 (0)	5 (19.2) 1 (20) 3 (60) 0 (0) 1 (20)	24 (20.3) 5 (20.8) 13 (54.2) 1 (4.2) 5 (20.8)	1 (4.3) 1 (100) 0 (0) 0 (0) 0 (0)	**< 0.001**
Routinely perform pre-operative endoscopy before Primary RYGB	202 (82.4)	41 (95.3)	18 (60)	19 (73.1)	108 (87.8)	16 (69.6)	**< 0.001**
Routinely perform post-operative endoscopy after Primary RYGB	45 (18.4)	14 (32.6)	5 (16.7)	7 (26.9)	12 (9.8)	7 (30.4)	**0.004**
Typical diameter of the gastrojejunostomy stoma	3 (2–3.75)	2.5 (2–3)	2.75 (2–3)	2.5 (2–3)	3 (2.5–4)	2.5 (2–3)	**0.024**
Type of gastrojejunostomy construction Circular stapler Hand-sewn anastomosis Linear stapler	10 (4.1) 36 (14.7) 199 (81.2)	0 (0) 3 (7) 40 (93)	1 (3.3) 7 (23.3) 22 (73.3)	1 (3.8) 7 (26.9) 18 (69.2)	8 (6.5) 13 (10.6) 102 (82.9)	0 (0) 6 (26.1) 17 (73.9)	**0.001**
Size of reload used in linear stapler (mm)	45 (30–45)	45 (30–45)	45 (30–60)	45 (35–60)	45 (35–45)	45 (12.1–60)	0.876
Size of reload used in circular stapler (mm)	25 (21–25)	-	25 (25–25)	-	25 (25–25)	-	0.100
Sutures used in hand-sewn anastomosis Barbed Non-barbed	19 (52.7) 17 (47.3)	0 (0) 3 (100)	5 (71.4) 2 (28.6)	1 (14.3) 6 (85.7)	9 (69.2) 4 (30.8)	2 (33.3) 4 (66.7)	**0.030**
Routinely close both the Petersen’s space and the Jejunojejunal mesenteric defect) Yes, all defects Yes, not all defects Jejunojejunal Mesenteric defect Petersen’s space No	169 (69) 49 (20) 34 (69.4) 15 (30.6) 27 (11)	27 (62.8) 10 (23.3) 9 (90) 1 (10) 6 (14)	20 (66.7) 8 (26.7) 5 (62.5) 3 (37.5) 2 (6.7)	18 (69.2) 5 (19.2) 4 (80) 1 (20) 3 (11.5)	87 (70.7) 20 (16.3) 12 (60) 8 (40) 16 (13)	17 (73.9) 6 (26.1) 4 (66.7) 2 (33.3) 0 (0)	0.600
Type of closure used to close the defects Barbed Sutures Non- Barbed Sutures Clips or Endo hernia Staplers Surgical Glue	97 (39.6) 98 (40) 12 (4.9) 5 (2.04)	6 (14) 29 (67.4) 1 (2.3) 0 (0)	8 (26.7) 17 (56.6) 2 (6.7) 0 (0)	15 (57.7) 6 (23.1) 0 (0) 4 (15.4)	65 (52.8) 25 (20.3) 10 (8.1) 2 (1.6)	6 (26.1) 16 (69.6) 0 (0) 0 (0)	**< 0.001**

Data are n (%) unless otherwise indicated; continuous variables are median (Q1–Q3). Ring diameter and gastrojejunostomy stoma diameter are reported in cm; limb lengths are reported in cm (biliopancreatic limb from DJ, alimentary limb from GJ, and common channel); bougie size is reported in French gauge (Fr). P-values for between-chapter comparisons were derived using Pearson’s χ² test for categorical variables when all expected cell counts were ≥ 5 or Fisher’s exact test when any expected cell count was < 5, and the Kruskal–Wallis test for continuous variables (two-sided). GJ: gastrojejunostomy

### Gastric Remnant

Routine resection of the gastric remnant during primary RYGB was reported by only one respondent (0.4%) (Table 7). No significant difference in remnant resection was observed between low-volume and high-volume surgeons (*p* = 1.000) (Table 5). Reinforcement of the gastric remnant was reported by 54 surgeons (22.0%), with clips being the most commonly used method (44.4%), followed by non-barbed sutures (25.9%), barbed sutures (18.5%), and other techniques (11.1%) (Table 7). Reinforcement methods differed significantly across IFSO chapters (*p* < 0.001) (Table 7). In contrast, both the rate of remnant reinforcement (31.7% vs. 26.7%, *p* = 0.968) and the distribution of reinforcement materials were similar between low- and high-volume surgeons (Table 5).

### Roux-en-Y Limb Lengths

The biliopancreatic limb (BPL) length was relatively consistent across respondents, with a global median of 100 cm (IQR: 77.5–120), and no significant differences between IFSO chapters (*p* = 0.103) (Table 4) or between low- and high-volume surgeons (*p* = 0.957) (Table 5). Only 53 surgeons (21.6%) reported routinely measuring the total bowel length (Table 4). In contrast, the alimentary limb (AL) length had a global median of 120 cm (IQR: 100–150) and differed significantly across IFSO chapters (*p* < 0.001) (Table 4), with the longest median length reported in NAC (150 cm) and shorter median lengths reported in LAC and APC (100 cm each). No significant difference in AL length was observed between volume groups (*p* = 0.839) (Table 5). The global median common channel (CC) length was 300 cm (IQR: 175–325) (Table 4), with no significant differences across IFSO chapters (*p* = 0.220) (Table 4) or between low- and high-volume surgeons (*p* = 0.333) (Table 5).

### Mesenteric Defect Closure

Routine closure of all internal mesenteric defects during primary RYGB was reported by 169 surgeons (69.0%), whereas 34 respondents (13.9%) reported closing only the jejunojejunal mesenteric defect, 15 (6.1%) closed only Petersen’s space, and 27 (11.0%) reported not closing any internal defects (Table 7). The overall pattern of defect closure did not differ significantly across IFSO chapters (*p* = 0.600) (Table 7) or between low- and high-volume surgeons (*p* = 0.261) (Table 5). In contrast, the technique used for defect closure varied significantly across IFSO chapters (*p* = 0.001) (Table 7). Barbed sutures and non-barbed sutures were the most commonly reported closure methods overall, whereas clips were used infrequently (Table 7). Closure technique did not differ significantly between low- and high-volume surgeons (*p* = 0.129) (Table 5).

### Esophagogastroduodenoscopy (EGD) practices

A preoperative esophagogastroduodenoscopy (EGD) was routinely performed by 82.4% of surgeons before primary RYGB, with significant variation across IFSO chapters (*p* < 0.001) (Table 7). Routine preoperative EGD was most frequently reported in LAC (95.3%) and least frequently in MENAC (60.0%) (Table 7). Although low-volume surgeons more often reported routine preoperative EGD than high-volume surgeons (88.3% vs. 75.0%), this difference was not statistically significant (*p* = 0.097) (Table 5). In contrast, routine postoperative EGD was reported by only 18.4% of surgeons, with significant regional variation (*p* = 0.004) (Table 7). No significant difference in postoperative EGD practice was observed between low- and high-volume surgeons (23.3% vs. 16.7%, *p* = 0.494) (Table 5).

### Intraoperative Leak Testing and Adjuncts

Routine intraoperative leak testing during primary RYGB was reported by 91.0% of respondents, with significant variation across IFSO chapters (*p* < 0.001) (Table 8). No significant difference in routine leak testing was observed between low- and high-volume surgeons (91.7% vs. 85.0%, *p* = 0.394) (Table 5). Endoscopic leak testing was less common, reported by 37 surgeons (15.1%), and also varied significantly across IFSO chapters (*p* = 0.005) (Table 8), whereas no significant difference was found between low- and high-volume surgeons (16.7% vs. 13.3%, *p* = 0.799) (Table 5).

**Table 8:** Use of intraoperative adjuncts and postoperative practices after primary RYGB, stratified by IFSO regional chapter

	Total *n* = 245	Latin America *n* = 43	Middle East-North Africa *n* = 30	Asia-Pacific *n* = 26	European *n* = 123	North American *n* = 23	*P*-value
Routinely insert a drain after Primary RYGB Yes On patient selection No	53 (21.6) 18 (7.3) 174 (71)	8 (18.6) 4 (9.3) 31 (72.1)	11 (36.7) 3 (10) 16 (53.3)	6 (23.1) 4 (15.4) 16 (61.5)	25 (20.3) 6 (4.9) 92 (74.8)	3 (13) 1 (4.3) 19 (82.6)	0.222
Routinely perform an intraoperative leak test in Primary RYGB	223 (91)	42 (97.7)	30 (100)	20 (76.9)	109 (88.6)	22 (95.7)	**< 0.001**
Routinely perform an Intraoperative endoscopic leak test	37 (15.1)	4 (9.3)	9 (30)	3 (11.5)	13 (10.6)	8 (34.8)	**0.005**
Routinely use indocyanine green (ICG) during RYGB	24 (9.8)	1 (2.3)	4 (13.3)	1 (3.8)	14 (11.4)	4 (17.4)	0.145
Typical length of typical hospital stays after RYGB in Days	2 (1–2)	1 (1–2)	1 (1–2)	2 (2–2.5)	2 (2–3)	1 (1–1)	**< 0.001**
Typical follow-up period after RYGB in Months	12 (3–36)	18 (12–24)	12 (12–27)	15 (2.5–36)	18 (3–60)	18 (12–36)	0.973
Routinely order a postoperative CT contrast study	10 (4.1)	0 (0)	2 (6.7)	3 (11.5)	4 (3.3)	1 (4.3)	0.181
Routinely order a postoperative barium X-ray	30 (12.2)	1 (2.3)	4 (13.3)	5 (19.2)	20 (16.3)	0 (0)	**0.037**

Data are n (%) unless otherwise indicated; continuous variables are median (Q1–Q3). LOS is reported in days; follow-up is reported in months. ICG, indocyanine green; CT, computed tomography. P-values reflect between-chapter comparisons using χ²/Fisher’s exact tests for categorical variables and Kruskal–Wallis tests for continuous variables (two-sided)

Use of indocyanine green (ICG) fluorescence imaging was uncommon, reported by 9.8% of respondents, with no significant variation across IFSO chapters (*p* = 0.145) (Table 8) or between volume groups (10.0% in both groups, *p* = 1.000) (Table 5). Drain placement was reported by 53 surgeons (21.6%), with no significant differences across IFSO chapters (*p* = 0.222) (Table 8) or between low- and high-volume surgeons (23.3% vs. 25.0%, *p* = 0.889) (Table 5).

### Postoperative Protocols

Routine postoperative imaging was infrequently used after primary RYGB. Only 4.1% of surgeons routinely requested computed tomography (CT) with contrast, whereas 12.2% reported using routine postoperative barium studies (Table 8). Use of postoperative barium studies differed significantly across IFSO chapters (*p* = 0.037), and was more common among low-volume than high-volume surgeons (23.3% vs. 3.3%, *p* = 0.002) (Tables 5 and 8). Routine CT use was low in both groups, with no significant difference by surgical volume (8.3% vs. 1.7%, *p* = 0.207) (Table 5).

The median length of hospital stay was 2 days (IQR: 1–2), with significant variation across IFSO chapters (*p* < 0.001) (Table 8). Low-volume surgeons reported a longer median hospital stay than high-volume surgeons (2 days [IQR: 1–3] vs. 1 day [IQR: 1–2], *p* = 0.002) (Table 5).

The reported duration of postoperative follow-up had a global median of 12 months (IQR: 3–36), with no significant difference across IFSO chapters (*p* = 0.973) (Table 8). However, high-volume surgeons reported significantly longer follow-up than low-volume surgeons (21 months [IQR: 12–60] vs. 12 months [IQR: 3–24], *p* = 0.042) (Table 5).

### Post Hoc Power Analysis

Post hoc power analysis demonstrated high achieved power for several representative between-region comparisons. Routine reinforcement of the gastric pouch showed a moderate-to-large effect size (Cohen’s w = 0.35, *p* = 0.002), with achieved power exceeding 99.7%. Routine pre-operative endoscopy demonstrated a moderate effect size (Cohen’s w = 0.30, *p* < 0.001), with power exceeding 97.7%. Preferred gastrojejunostomy construction technique also showed a moderate effect size (Cohen’s w = 0.33, *p* = 0.001), corresponding to achieved power exceeding 99.3%.

## Discussion

### RYGB: Technical Variation Across Several Operative Steps

Although common reconstructive principles define RYGB, our survey shows that technical variation persists across several operative steps. These findings highlight incomplete standardization in reported practice and may complicate comparisons across studies and registries, particularly when key technical details are inconsistently defined or reported [23, 24]. Although international surveys have previously documented such variation [23, 24], our data provide an updated international snapshot and reinforce the need for clearer operational definitions of key technical steps. However, the present survey was not designed to determine which differences are clinically consequential and which may reflect acceptable technical preference. Accordingly, the main implication of these findings is not that all variation should be eliminated, but that more consistent technical reporting is needed to support future outcome-linked analyses and consensus development [25–28]. A unified operational definition of “standard” RYGB remains absent, and technical choices may still be influenced by training background and local norms rather than harmonized protocols [29–32].

### Pouch Configuration: Under-Measured, Over-Described

Gastric pouch configuration may influence outcomes after RYGB, including weight loss, reflux control, and marginal ulceration (MU) [17, 33–36]. In this survey, many surgeons subjectively described pouch size, with 67.3% labeling it as short or small, while only 53.5% measured pouch length. Termination landmarks varied, with 53.9% reporting division between the first and middle gastric veins. This indicates that terms like “short” and “long” lack consistent anatomical definitions. Higher surgical volume did not significantly reduce variability in pouch measurement; both low- and high-volume surgeons reported similar median pouch lengths. Though high-volume surgeons more often described their pouches as “long,” this was not statistically significant.

This lack of standardized reporting complicates the interpretation of the broader literature. Prior studies have linked pouch dimensions to outcomes such as MU and weight-loss durability, but comparisons remain difficult because pouch size is reported using heterogeneous proxies, including subjective descriptors, stapler-based estimates, anatomic landmarks, and non-uniform volumetric measures [17, 37–42]. Some reports link a larger pouch size to higher MU risk; for example, Ayuso et al. reported increasing MU risk with increasing pouch volume [43], while other data suggest pouch size may also interact with weight-loss durability [33, 37, 38, 43, 44]. Without consistent, reproducible measurement, these associations remain hard to compare and translate. Accordingly, our findings support the value of more consistent reporting of pouch length in centimeters, together with clearly defined termination landmarks and calibration details when used.

### Bougie Calibration

Bougie (calibration tube) size affects pouch diameter and restriction and may influence satiety, weight loss, and technical risk. In our survey, 36 Fr was the most common size globally, but practice ranged from 28 to 46 Fr with significant regional variation. However, most reported sizes clustered within a relatively narrow range, predominantly between 32 and 40 Fr. Bougie size distribution did not differ by surgical volume, and bougie size was often not accompanied by objective pouch measurements, limiting inference about its clinical impact.

Unlike sleeve gastrectomy, where bougie effects are well studied [45, 46], RYGB-specific evidence is comparatively limited [47]. However, some RYGB-specific studies suggest that calibration strategy and pouch size may influence postoperative weight loss, although the evidence remains limited and does not establish an optimal bougie caliber [34, 47]. Accordingly, the variation observed here is more appropriately interpreted as reflecting modest technical preference rather than clearly distinct procedural constructs. A practical implication is that bougie size should be reported together with measured pouch dimensions, enabling clearer evaluation of technique–outcome relationships in future studies.

### Limb Lengths: Regional Variation Within Predominantly Fixed-Length Practice

Limb configuration (BPL, AL, and CC) is an important technical component of RYGB [37, 48–50]. Yet measurement-based tailoring remains uncommon: only 21.6% of respondents reported routinely measuring total bowel length. In this context, it is unsurprising that practice clusters around convention rather than individualized allocation. In our dataset, BPL lengths clustered tightly (little regional separation), whereas AL length showed the greatest regional variation.

Salminen and Peterli (2021) emphasized the importance of considering BPL, CC, and total alimentary limb length (TALL) as interdependent variables, particularly because excessively short CC (< 200 cm) or TALL (< 400 cm) may increase the risk of protein malnutrition [50]. Randomized evidence from the Norwegian distal RYGB series and the DUCATI trials has shown mixed short-term findings, whereas longer-term DUCATI follow-up suggested more durable weight-loss benefit with longer ALs (> 150 cm) without increased malnutrition or mortality [51, 52]. Taken together, these data support evaluating limb configuration as a composite construct rather than as isolated segment lengths.

In our survey, both low- and high-volume surgeons most commonly reported fixed-length configurations, and routine total bowel measurement remained uncommon. This finding should be interpreted cautiously, however, because bowel measurement itself is not uniformly standardized in clinical practice and may be influenced by technical preference, operative context, and concerns about bowel handling. Accordingly, the present survey does not allow conclusions about whether broader use of measurement-based tailoring would be preferable, but it does highlight ongoing variation in how limb configuration is approached in routine practice.

### Gastrojejunostomy and Stoma Size: Uniform Tools, Divergent Techniques

The gastrojejunostomy (GJ) is a key determinant of RYGB outcomes, affecting stricture risk, dumping, marginal ulceration (MU), and weight trajectory [44, 53, 54]. Our survey revealed significant variation in practice, with linear stapled anastomosis (LSA) being the most common technique (81.2%), followed by hand-sewn (14.7%) and circular stapled (4.1%). Technique choice varied by region, indicating that GJ selection is more influenced by training and local practices than a standardized approach. There was also significant variation in stoma sizing, with a median diameter of 3.0 cm, but no clear standardization in technique or size was observed.

This variation is clinically relevant. Smaller stoma sizes may promote early satiety and enhanced restriction, but are associated with a higher risk of anastomotic stricture [53]. Larger diameters may reduce stricture rates but increase the risk of dumping and RWG [53–55]. Furthermore, the type of suture material may influence the development of MUs. Conditions that promote local inflammation, such as non-absorbable sutures, staple line tension, and persistent foreign body contact, have been implicated in MU formation [44, 56, 57]. Palermo et al. demonstrated that MU incidence decreased from 2.6% with non-absorbable sutures to 1.3% with absorbable sutures [58]. The technique of GJ construction also impacts MU incidence. CSA has been associated with higher MU rates compared to LSA and HAS [59–62]. Lois et al. reported MU rates of 5.5% for CSA versus 0.7% for HSA [62], while Major et al. found 10.3% for CSA compared to 2.1% for LSA [61].

Volume-based subgroup analysis did not show statistically significant differences in stoma diameter or preferred gastrojejunostomy technique, suggesting that surgical volume alone does not ensure convergence in this aspect of RYGB construction. Nevertheless, the observed diversity in technique and sizing continues to support more consistent reporting of anastomotic configuration, suture material, and stoma dimensions in future studies and registries.

### Mesenteric Defect Closure: Broad Adoption, Fragmented Techniques

Internal hernia (IH) is a serious late complication of RYGB, linked to bowel obstruction, chronic pain, and ischemia [63–66]. While mesenteric defect closure, especially at the jejunojejunostomy and Petersen’s spaces, is widely endorsed to reduce IH risk [65–67], our data show variations in techniques and materials used [68–70]. Among respondents, 89% reported routine closure, with 69% closing both key defects, but 20% closed selectively, and 11% did not close any defects, particularly in LAC and EC settings.

No significant volume-based difference was observed in the overall pattern of defect closure. Randomized evidence supports routine closure to reduce IH, but concerns like early obstruction and reintervention have been noted [71]. Various techniques, including absorbable sutures, clips, staples, and tissue glue, aim to balance efficacy and safety [66, 67]. However, no head-to-head trials exist comparing these methods, and most registries do not systematically document closure techniques or materials.

Given the clinical relevance of defect closure, the observed variation in closure strategies warrants further outcome-linked evaluation. The present survey identifies heterogeneity in both whether and how defects are closed, but it was not designed to determine the optimal closure method. Until closure techniques are standardized and linked to outcomes in registry-based datasets, practice will remain variable and data-inconclusive.

### Ring Augmentation

Ring augmentation (raRYGB) was used by a minority of respondents in our cohort (20.4%). Its use varied across regions and was not reported by respondents from NAC, suggesting that adoption remains heterogeneous across practice settings [18, 30, 72–75]. Published studies [18, 30, 72–76] and the recent IFSO GRADE position statement [77] suggest that ring augmentation may provide superior or comparable weight-loss outcomes in selected settings, with generally low ring-related complication and removal rates, and cautiously support selective use, particularly for raRYGB. However, the literature remains heterogeneous and debated, and its interpretation requires caution.

This uncertainty is illustrated by the 2025 randomized controlled trial by Okkema et al., which found no significant long-term weight-loss advantage with the addition of a non-adjustable silicone ring and reported ring removal in 12% of patients because of dysphagia over five years [73]. Accordingly, the current evidence suggests that ring augmentation may offer benefit in some settings, but that this potential must be weighed against device-related symptoms and the possibility of later removal.

In this study, ring use was not associated with surgeon volume or annual caseload, but was significantly associated with years of experience in MBS, suggesting that familiarity and accumulated practice exposure may influence adoption more than procedural volume alone. Among ring users, both peri-gastric and pars flaccida approaches were reported, with regional variation in preferred technique. Reported ring diameter was generally consistent, and no clear correlation was observed between ring diameter and bougie size, indicating that ring-related decisions may be made independently of other technical components of the procedure [18, 73, 78, 79].

Taken together, these findings indicate that ring augmentation remains selectively adopted in contemporary RYGB practice. However, the present study was not designed to determine whether lower uptake reflects underuse, appropriate caution, selective indication, or local training preferences. Accordingly, our findings should be interpreted as descriptive of current reported practice rather than as evidence for broader or narrower adoption. Further comparative outcome-linked studies, with standardized reporting of ring type, technique, dysphagia, and ring removal, would be needed to clarify the role of ring augmentation across different RYGB constructs.

### Intraoperative Adjuncts: Leak Testing, ICG, and Drain Use

Intraoperative leak testing (ILT) was widely reported in our survey, with 91.0% of respondents incorporating it into primary RYGB. The most commonly used methods were air under water and methylene blue instillation. Despite its widespread adoption, the clinical value of routine ILT remains debated. Prior studies have suggested that ILT may help identify intraoperative technical problems, but its effect on postoperative leak-related outcomes remains uncertain [80, 81]. Nevertheless, its continued routine use in bariatric practice likely reflects its perceived value as an immediate intraoperative safety check, even if this benefit is not fully captured by postoperative outcome data.

In contrast, advanced adjuncts were used less frequently. Endoscopic leak testing and indocyanine green (ICG) fluorescence imaging were both uncommon in our survey, with routine ICG use reported by only 9.8% of respondents. ICG offers real-time perfusion assessment and may assist in evaluating pouch and anastomotic vascularity [82–85]. However, its uptake remains limited, likely owing to cost, workflow demands, resource availability, and the need for technical familiarity. The present study was not designed to determine whether broader adoption of ICG or endoscopic adjuncts would improve outcomes, but the observed variation suggests that their use remains selective rather than routine.

Drain placement was also selective rather than routine, reported by 21.6% of surgeons in our cohort. Evidence regarding prophylactic drains in MBS remains inconclusive, and several studies suggest that routine drainage may not reduce leak-related morbidity while potentially increasing discomfort, prolonging hospitalization, or adding procedure-related burden [80, 81]. In this context, the observed variation in drain use likely reflects differences in surgeon preference and institutional practice rather than a clearly established standard. Overall, our findings indicate that while ILT is commonly embedded in routine RYGB practice, the use of ICG and drains remains more variable and selectively applied.

### Surgical Volume and Outcomes: Differences in Follow-up and Resource Use Rather Than Clear Technical Convergence

Higher procedural volume was associated with shorter reported hospital stay and longer follow-up duration, whereas core intraoperative practices differed little between high- and low-volume surgeons. Linear stapling remained the predominant gastrojejunostomy technique in both groups, and both largely favored fixed-length limb configurations without significant differences in reported bowel lengths. Routine leak testing was common regardless of volume, while endoscopic leak testing, ICG use, and drain placement remained uncommon in both groups. By contrast, low-volume surgeons more often reported routine postoperative imaging, particularly barium studies. Overall, these findings suggest that surgical volume in this survey was associated more with differences in follow-up organization and postoperative surveillance than with clear technical convergence, although the study was not designed to determine the clinical significance of these differences.

### Strengths and Limitations

To our knowledge, this study represents one of the most comprehensive mappings of the RYGB technique to date, surveying 245 MBS surgeons from 47 countries across all IFSO chapters. It provides an in-depth overview of global variations in procedural choices, including pouch size and anastomotic techniques. Unlike previous reports focused on procedure counts, this work highlights key technical variables that may influence postoperative outcomes but are often missing from global registries.

However, several limitations should be considered: self-reporting may introduce recall and social-desirability bias; response distribution was uneven, with underrepresentation from NAC and overrepresentation from EC; and the absence of linked postoperative outcomes precludes causal inferences regarding effectiveness. In addition, the sampling frame was restricted to surgeons with available and deliverable contact details, and the high rate of undeliverable emails suggests potential coverage and nonresponse bias, limiting generalizability to the full IFSO membership. Invitations were sent to all retrieved contacts; however, delivery status was not systematically analyzed by region, and respondents were grouped by IFSO chapter only after survey completion. Therefore, regional equality in the delivery of invitations could not be confirmed retrospectively.

An additional limitation is that antecolic versus retrocolic reconstruction was not captured in the survey, despite being a relevant technical component of RYGB. Future survey-based studies and technical reporting frameworks should consider including this variable. Although post hoc power analysis suggested adequate statistical power to detect moderate regional differences in several key variables, the sample may still be insufficient to precisely characterize rare practices or to support stable estimates in smaller regional subgroups, particularly in chapter-level analyses.

Despite these limitations, the study’s scope and technical granularity provide a useful foundation for prioritizing a minimal technical dataset and informing future standardization and registry initiatives.

### Technical Variables Remain Underrepresented in Registries

Although major international registries increasingly capture patient characteristics, procedure types, and short-term outcomes, detailed operative technique remains inconsistently recorded. Variables such as pouch length, bougie size, limb configuration, stoma diameter, and use of reinforcement or adjuncts are often absent.

This limits the ability to examine how technical variation may relate to long-term weight, metabolic, nutritional, and complication outcomes. At the same time, not all technical differences are likely to carry equal clinical importance, and outcome-linked studies will be needed to distinguish major from minor sources of heterogeneity. Our findings, therefore, highlight not only the extent of variation in reported practice but also the persistent gap in technical data capture that constrains data-driven refinement of RYGB.

### Call to Action

To bridge the gap between surgical variation and patient outcomes, professional societies and registry leaders should prioritize the standardization and systematic capture of key intraoperative technical variables. At a minimum, this includes routine documentation of pouch length, bougie size, limb lengths, anastomotic configuration, ring augmentation details, and the technique and materials used for internal defect closure. These variables should be incorporated into global MBS datasets and registries. Societies like IFSO and regional affiliates may also help by developing technical checklists to harmonize surgical reporting. Although complete uniformity in RYGB technique may not be achievable, more consistent reporting is a practical first step toward understanding which technical variations are clinically meaningful and which reflect acceptable procedural preference.

### Future Direction and Ideas for Further Research

The design and implementation of randomized controlled trials in a multicenter setting would offer valuable information and data to identify the most effective, reproducible, safe, and cost-effective variations and techniques to perform RYGB across multiple chapters of IFSO. In addition, surgical education and training courses should be prioritized at IFSO and its chapter conferences to promote more consistent technical understanding and reporting among members.

## Conclusion

RYGB remains one of the most frequently performed MBS procedures worldwide, yet its technical execution varies significantly. This global survey reveals diverse practices in core operative steps such as pouch configuration, limb lengths, stoma calibration, and defect closure. Collectively, these findings indicate that important technical variation exists in the performance of primary RYGB across surveyed surgeons. However, given the descriptive design and lack of outcome-linked data, the present study cannot determine which variations are clinically consequential or which approach should be preferred.

At present, routine registry capture of detailed technical variables remains limited, which constrains efforts to evaluate how these differences may relate to long-term efficacy and safety. To address this issue, standardized reporting of key technical parameters should be integrated into surgical registries, and international societies should create technical reporting frameworks. Only through consistent documentation can we start to understand which of these variations have meaningful clinical implications and which may reflect acceptable technical preference without major outcome impact. Progress toward global consensus in RYGB begins with knowing the specifics of our approaches.

## Data Availability

De-identified survey data and analysis scripts will be made available by the corresponding author upon reasonable request.
